# Translation control of Enterovirus A71 gene expression

**DOI:** 10.1186/s12929-019-0607-9

**Published:** 2020-01-08

**Authors:** Ming-Chih Lai, Han-Hsiang Chen, Peng Xu, Robert Y. L. Wang

**Affiliations:** 1 Department of Biomedical Sciences, College of Medicine, Chang Gung University, Taoyuan, 33302 Taiwan; 2 Graduate Institute of Biomedical Sciences, College of Medicine, Chang Gung University, Taoyuan, 33302 Taiwan; 3 Department of Colorectal Surgery, Chang Gung Memorial Hospital at Linkou, Taoyuan, 33305 Taiwan; 4 Xiangyang No.1 People’s Hospital, Hubei University of Medicine, Shiyan, Hubei Province China; 5 Division of Pediatric Infectious Disease, Department of Pediatrics, Chang Gung Memorial Hospital at Linkou, Taoyuan, 33305 Taiwan

**Keywords:** Enterovirus A71, Translational control, Host factors, Gene expression

## Abstract

Upon EV-A71 infection of a host cell, EV-A71 RNA is translated into a viral polyprotein. Although EV-A71 can use the cellular translation machinery to produce viral proteins, unlike cellular translation, which is cap-dependent, the viral RNA genome of EV-A71 does not contain a 5′ cap and the translation of EV-A71 protein is cap-independent, which is mediated by the internal ribosomal entry site (IRES) located in the 5′ UTR of EV-A71 mRNA. Like many other eukaryotic viruses, EV-A71 manipulates the host cell translation devices, using an elegant RNA-centric strategy in infected cells. During viral translation, viral RNA plays an important role in controlling the stage of protein synthesis. In addition, due to the cellular defense mechanism, viral replication is limited by down-regulating translation. EV-A71 also utilizes protein factors in the host to overcome antiviral responses or even use them to promote viral translation rather than host cell translation. In this review, we provide an introduction to the known strategies for EV-A71 to exploit cellular translation mechanisms.

## Background

Enterovirus A71 (EV-A71) infection is one of the major causes of hand-foot-and-mouth disease (HFMD) mainly in young children and infants worldwide [1, 2]. In 1969, EV-A71 was first isolated from a child with encephalitis in California, USA [1]. Since then, several outbreaks have been reported worldwide. In 1998, EV-A71 infection caused HFMD and herpangina in more than one hundred thousand people in Taiwan, which led to 78 fatalities because of serious neurological complications [2]. EV-A71 epidemic has therefore become a serious threat to public health, especially in the Asia-Pacific region. EV-A71 transmits mainly via oral-fecal aerosol and droplet routes [3]. To date, human is the only known host found for EV-A71. Generally, EV-A71 infection is asymptomatic to a mild form of disease and can be overcome by our immune system. The typical clinical symptoms of HFMD caused by EV-A71 include papulovesicular which is the rash found on hands and legs and herpangina which are the ulcers on lips and tongue [3]. Occasionally, EV-A71 can invade into central nervous system (CNS) to cause acute neurological complications, such as aseptic meningitis and encephalitis, and even lead to death. Although EV-A71 is a neurotropic virus, the chance is not high for it to invade into CNS due to the existence of human physical barriers, such as blood brain barrier (BBB). Viruses transmit into CNS via BBB route, which is mediated by immune cells or via retrograde axonal transport. Retrograde axonal transport has been reported to be the major route for EV-A71 to invade into CNS in mice. However, as EV-A71 can infect immune cells, BBB route is also possible to be employed by EV-A71 to facilitate its invasion into CNS. Both EV-A71 replication in CNS and the subsequent cytokine storm caused by the over stimulation of the immune system might contribute to the acute neurological complications. Overall, in addition to HFMD, EV-A71 can also cause diverse neurological complications, such as aseptic meningitis, poliomyelitis-like acute flaccid paralysis, brainstem encephalitis, or even death [3]. In the past, there have been quite a few literatures on molecular biology research on EV-A71, from viral entry, viral replication, viral packaging, and the relationship between viruses and hosts [4]. In this review, we focus on the advances in translational control of EV-A71 gene expression. A better understanding of the regulation of viral gene expression will certainly contribute to the development of vaccines and antiviral drugs.

## Structure and function of Enterovirus A71 viral RNA and proteins

**Fig. 1:**
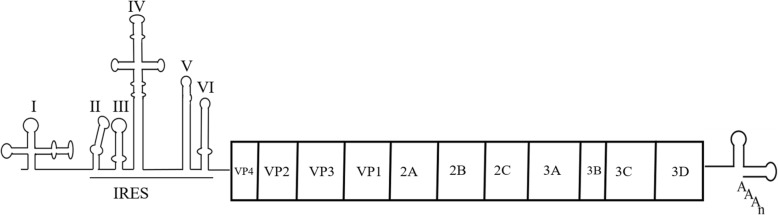
The structure of the EV-A71 genome. The 5′ UTR of EV-A71 contains six stem-loop RNA domains (I-VI). Domain I functions in negative-strand RNA synthesis, whereas domains II–VI form an IRES element. The ORF encodes a polyprotein, which is cleaved into 11 viral proteins by viral and/or cellular proteases

EV-A71 is a small non-enveloped virus composed of an icosahedral capsid and belongs to the genus of *Enterovirus* in the *Picornaviridae* family. The structure of the EV-A71 genome is a single-stranded positive-sense RNA encoding a large open reading frame (ORF) flanked by a highly structured 5′-untranslated region (UTR) and a 3′-UTR with a poly (A) tail (Fig. 1). The 5′-UTR of EV-A71 RNA is approximately 750 nucleotides (nt) that contains six stem-loop structures (domains I-VI) [5]. The cloverleaf-like domain I is a cis-acting replication element for the synthesis of negative strand RNA as the template [6], whereas domains II-VI form an internal ribosome entry site (IRES) element that facilitates ribosome recruitment. EV-A71 RNA lacks the 5′ cap structure (m7GpppN) and thus initiates translation of the viral RNA by a cap-independent and IRES-mediated mechanism [7]. Viral RNA encodes a large polyprotein that, through a series of proteases-mediated processing events, produces 11 viral proteins, including 4 structural proteins (VP1, VP2, VP3, and VP4) and 7 non-structural proteins (2A, 2B, 2C, 3A, 3B, 3C, and 3D). The non-structural proteins are involved in the expression and replication of viral genes. The 3′-UTR of EV-A71 RNA contains three putative stem-loop structures (X, Y, and Z) that are involved in viral replication [8]. In order to complete the life cycle of EV-A71, viral proteins are involved in translational control of viral and host mRNAs.

## Inhibition of host cell translation after EV-A71 infection

EV-A71 causes rapid inhibition of host cell cap-dependent translation during viral infection, and this preferably allows for cap-independent translation of its own genomic RNA by the IRES element [9]. EV-A71-encoded proteases 2A^pro^ and 3C^pro^ are important for viral polyprotein processing. Viral proteases not only cleave viral polypeptides, but also inhibit cap-dependent translation primarily by cleavage of translation initiation factors (eIFs) in host cells. 2A^pro^ cleaves eukaryotic initiation factor 4G (eIF4G) [10–14], 3C^pro^ cleaves eukaryotic initiation factor 4A (eIF4A) [15] and eukaryotic initiation factor 5B (eIF5B) [16], resulting in the shut off of host cell translation. Cleavage of poly (A)-binding protein (PABP) by 3C^pro^ also helps to inhibit host cell translation [17–19]. In addition, 2A^pro^ induces stress granule formation in the EV-A71-infected cells [20]. Stress granules formation is accompanied by disassembly of polysomes and translation inhibition [21]. EV-A71 infection also induces endoplasmic reticulum (ER) stress [22]. Under such a condition, the double-stranded RNA-dependent protein kinase PKR phosphorylates the regulatory α subunit of eukaryotic translation initiation factor 2 (eIF2α) to block translation of both cellular and viral mRNAs. After EV-A71 infection, 3C^pro^ cleaves PKR to activate viral translation and replication [23]. Notably, a cleavage fragment of eIF5B, a product of viral 3C^pro^, can be substituted for eIF2 to deliver Met-tRNAi to the 40S ribosomal subunit, while eIF2α is phosphorylated and inactivated by viral infection [24]. Therefore, the regulation of EV-A71 mRNA translation may be a dynamic process.

## Mechanism of EV-A71 viral RNA translation

**Fig. 2:**
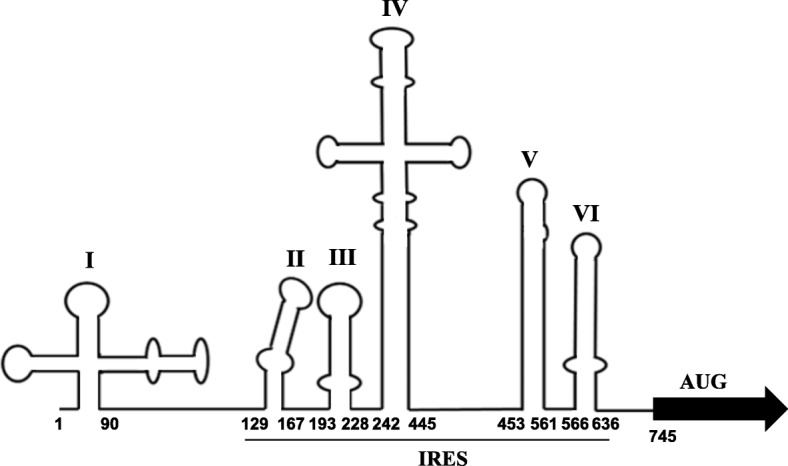
Diagram of the EV-A71 5′ UTR. Line drawing shows the predicted secondary structure motifs. The first and last nucleotides in each stem-loop domains are numbered. Domain II to VI constitute the IRES element

Translation of EV-A71 is mediated by a type 1 IRES element in the 5′-UTR of viral RNA, allowing proceed the cap-independent of viral protein synthesis in the host cells [7]. EV-A71 IRES (domains II-VI) spans approximately 450 nt long (Fig. 2). Domain II is a short stem-loop that harbors a conserved AUAGC motif. Domains III and VI are more variable, whereas domains IV and V are relatively conserved. Domain IV harbors an internal C-rich loop and a GNRA (N stands for any nucleotide, and R for purine) motif [25]. Domain V consists of a hairpin with an internal loop and interacts with eIF4G and eIF4A for 48S ribosomal assembly [26]. The Yn-Xm-AUG (Yn is a pyrimidine-rich region and Xm is a 15- to 25-nucleotide spacer followed by an AUG codon) motif is conserved in most picornaviruses and located within domain VI of the IRES element. This motif has been proposed to be the ribosome entry site but not as a translation initiation codon [27]. The real AUG start codon is about 750 nt downstream of the 5′ end. The translation of IRES for EV-A71 still requires binding of the canonical initiation factors, including eIF1, eIF1A, eIF2, eIF3, eIF4A, eIF4B, and the central domain of eIF4G [28]. The truncated eIF4G is a product of the viral 2A^pro^ that specifically binds to domain V of EV-A71 IRES and recruits eIF4A to promote the formation of the 43S pre-initiation complex [26]. In contrast, hepatitis C virus (HCV) IRES is the prototype of type 3 IRESs that requires only a small portion of the canonical initiation factors to form the 48S initiation complex [29]. Many viral IRESs require a variety of RNA binding proteins (RBPs), termed IRES-transacting factors (ITAFs), to facilitate the recruitment of the 40S ribosomal subunits [30]. To date, many ITAFs have been shown to stimulate EV-A71 IRES activity, including heterogeneous nuclear ribonucleoprotein A1 (hnRNP A1) [31–33], polypyrimidine tract-binding protein 1 (PTB1) [34–37], poly (rC)-binding proteins 1 and 2 (PCBP1/2) [38–40], the 68-kDa Src-associated protein in mitosis (Sam68) [41], the DEAD-box RNA helicase DDX3 [42], HuR and Ago2 [43], and far-upstream element-binding protein 1 (FBP1) [44]. Although FBP1 was identified as an ITAF [44], FBP2 was shown to inhibit EV-A71 IRES activity in the EV-A71-infected cells [45]. AU-rich element-binding protein 1 (AUF1) also negatively regulates enterovirus infections [46, 47]. AUF1 and hnRNP A1 compete for the same IRES domain to downregulate or upregulate viral translation. Like many other viruses, EV-A71 translation is delicately regulated by host cell proteins.

## Cellular proteins involved in the regulation of EV-A71 mRNA translation

**Fig. 3:**
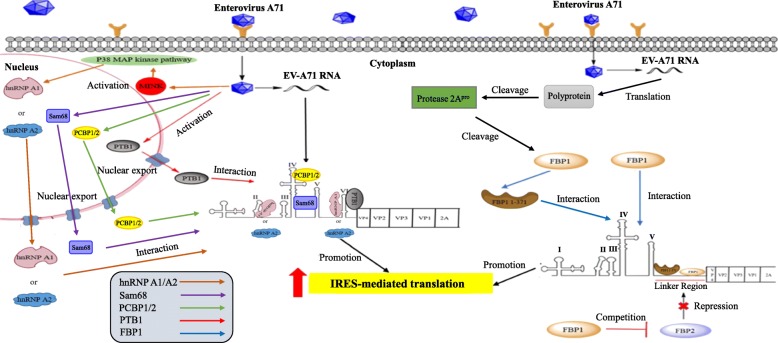
The regulatory roles of cellular IRES trans-acting factors (ITAFs) in EV-A71 translation. The brown arrow indicates that MINK is phosphorylated after EV-A71 infection. Phosphorylation of MINK activates p38 MAPK kinase pathway, which stimulates the export of hnRNP A1 from the nucleus into the cytoplasm, where hnRNP A1 binds to domains II and VI of EV-A71 IRES and then recruits the ribosome to promote viral IRES-mediated translation. Similarly, hnRNP A2 can replace hnRNP A1 to promote viral IRES-mediated translation. EV-A71 infection also activates nuclear Sam68, PCBP1/2, and PTB1 proteins to redistribute to the cytoplasm. Sam68, PCBP1/2, and PTB1 bind to different domains of EV-A71 IRES to promote viral translation. EV-A71 viral proteinase 2A^pro^ can cleave FBP1 to generates a functional cleavage product, FBP1^1–371^, and the cleavage product also acts to promote viral IRES-mediated translation. FBP1^1–371^ acts additively with FBP1 to promote IRES-mediated translation and virus production. FBP1 activates viral IRES activity by competing with FBP2, which also binds to EV-A71 IRES and acts as a negative regulator of EV-A71 translation

In EV-A71-infected cells, cap-dependent translation is shut off and IRES-mediated translation is activated by host cell proteins (Table 1). IRES elements can recruit the 40S ribosomal subunit directly or by using eIFs and auxiliary RBPs, which are identified as ITAFs. Most ITAFs are nuclear proteins that are redistributed to the cytoplasm during viral infection and cellular stress. After EV-A71 infection, many cellular proteins are attracted to the IRES element to facilitate viral mRNA translation (Fig. 3). Misshapen NCK-related kinase (MINK) is involved in many important cellular processes, such as cell growth, cytoskeletal rearrangement, and movement. At early stage of EV-A71 infection, EV-A71 induces phosphorylation of MINK, and the downstream of p38 MAPK, which then stimulates the relocalization of hnRNP A1 into the cytoplasm where it binds to the viral IRES and recruits ribosomes to promote IRES-mediated translation of viral mRNAs [55]. The hnRNP A1 binding sites on the EV-A71 IRES were identified in domains II and VI [32]. In addition, the function of hnRNP A1 in the enhancement of EV-A71 IRES-mediated translation can be substituted by hnRNP A2 (Fig. 3). When hnRNP A2 is inhibited, EV-A71 translation is reduced. It is found that hnRNP A2 interacts with EV-A71 IRES structure. Both hnRNP A1 and hnRNP A2 can synergistically promote the IRES-mediated translation of EV-A71. PTB1, also known as hnRNP I, was reported as an ITAF soon after the discovery of viral IRES [56]. PTB1 binds to pyrimidine-rich RNA sequences and has multiple functions in pre-mRNA splicing, polyadenylation, and viral IRES-mediated translation. PTB1 is involved in many viral translation initiations such as poliovirus, EMCV, HRV14, and FMDV. After EV-A71 infection, nuclear PTB1 is redistributed to the cytoplasm and interacts with domain VI of EV-A71 IRES via its RNA recognition motifs 1 and 2 (RRM1 and 2), thus increases the activity of EV-A71 IRES-mediated translation [34, 35] (Fig. 3). Sam68 is a 68 kDa nuclear protein associated with Src in mitosis, a member of the STAR family of proteins involved in message transmission and RNA activation. The cellular factor Sam68 binds specifically to EV-A71 IRES domains IV and V and acts as an ITAF to up-regulate viral translation [41] (Fig. 3). PCBP1/2 also function as ITAFs by interaction with domain IV of type 1 IRES to promote viral translation [48–50]. When PCBP1/2 is inhibited, IRES-mediated translation is reduced [57]. PCBP1/2 contain three hnRNP K homology (KH) domains which are involved in RNA binding [58]. PCBP2 binding to domain IV of EV-A71 IRES is also required for 48S complex formation and viral translation [28] (Fig. 3). The Ser-Arg-rich (SR) proteins is required for constitutive and alternative splicing. A subset of SR proteins shuttles continuously between the nucleus and the cytoplasm and play a role in mRNA translation [59]. It has been reported that SRp20 interacts with PCBP2 and functions to promote type 1 IRES-mediated translation [51, 52]. Thus, SRp20 may also function in facilitating EV-A71 translation. EV-A71 viral proteinase 2A^pro^ can cleave FBP1 to generates a functional cleavage product, FBP1^1–371^, and the cleavage product also acts to promote viral IRES-mediated translation [60] (Fig. 3). FBP1 binds to the EV-A71 5′ UTR linker region at nt. 686–714, while FBP1^1–371^ similarly binds to the 5′ UTR linker region at a different site located at nt. 656–674, and acts additively with FBP1 to promote IRES-mediated translation and virus production. Studies have already confirmed that most ITAF can enhance viral IRES activity; however, several ITAFs can repress IRES-mediated translation. FBP1 and FBP2 are two new ITAFs of EV-A71. Upon EV-A71 infection, FBP1 activates viral IRES activity by competing with FBP2, which also binds to the IRES of EV-A71 and acts as a negative regulator of EV-A71 translation [44, 45] (Fig. 3).

**Table 1:** Cellular proteins involved in the regulation of enterovirus A71 mRNA translation

Host factors	The effects of host proteins on EV-A71 translation	Refs
hnRNP A1/A2	hnRNP A1 promotes IRES-mediated translation after EV-A71 infection and its function in translation can be replaced by hnRNP A2.	[31–33]
PTB1	Nuclear PTB1 is transferred to the cytoplasm and interacts with EV-A71 IRES domain VI to promote viral mRNA translation.	[34–37]
PCBP1/2	PCBP1/2 also function as ITAFs by interaction with domain IV of type 1 IRES to promote viral translation.	[48–50]
Sam68	Sam68 binds specifically to EV-A71 IRES domains IV and V and acts as an ITAF to upregulate viral translation.	[41]
DDX3	DDX3 may indirectly binds to the domain VI of EV-A71 IRES and then unwind the secondary structure to facilitate ribosome entry.	[42]
HuR	HuR binds to the domain II of EV-A71 IRES by viral small RNA and promotes viral IRES-mediated translation.	[43]
Ago2	Ago2 binds to the domain II of EV-A71 IRES by viral small RNA and promotes viral IRES-mediated translation.	[43]
FBP1	FBP1 binds to the EV-A71 5′ UTR linker region to promote IRES-mediated translation and virus production. FBP1 activates viral IRES activity by competing with FBP2.	[44]
FBP2	FBP2 binds to the IRES of EV-A71 and acts as a negative regulator of viral IRES-mediated translation.	[45]
AUF1	AUF1 binds to the domain II of EV-A71 IRES by viral small RNA and represses viral IRES-mediated translation.	[46, 47]
SRp20	SRp20 interacts with PCBP2 and functions to promote type 1 IRES-mediated translation.	[51, 52]
Hsp27	Hsp27 activates viral protease 2A^pro^ to cleave host eIF4G protein, and thus inhibits host cap-dependent translation and enhances viral IRES-mediated translation.	[53]
Hsc 70	Hsc70 enhances viral 2A^pro^ activity to cleave host eIF4G protein, and thus inhibits host cap-dependent translation and enhances viral IRES-mediated translation.	[54]
MINK	EV-A71 infection induces the phosphorylation of MINK and then stimulates the transfer of hnRNP A1 into the cytoplasm where it binds to the viral IRES and promotes viral IRES-mediated translation.	[55]

**Fig. 4:**
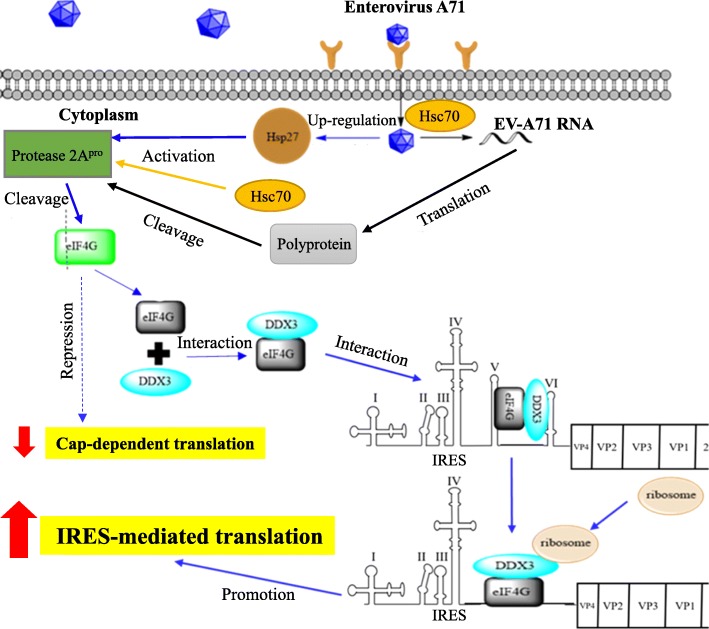
The regulatory roles of Hsp27, Hsc70, and DDX3 in EV-A71 translation. EV-A71 infection upregulates Hsp27 protein expression. Hsp27 can activate EV-A71 2A^pro^ to cleave eIF4G, leading to inhibition of cap-dependent translation. Hsc70 also activates EV-A71 2A^pro^ protein to cleave eIF4G and thus represses cap-dependent translation of host mRNAs. DDX3 interacts with the C-terminal cleavage fragment of eIF4G and binds to the domain VI of EV-A71 IRES. DDX3 may unwind RNA secondary structures to facilitate ribosome entry and thus enhance viral IRES-mediated translation

It has been well known that the proteolytic activity of viral 2A^pro^ is important for inhibiting host cap-dependent translation and enhancing viral IRES-mediated translation [61]. Viral 2A^pro^ cleaves host eIF4G protein to generate two fragments. The N-terminal cleavage fragment of eIF4G contains the binding site for eIF4E, leading to inhibition of cap-dependent translation. The C-terminal cleavage fragment of eIF4G is sufficient to promote IRES-mediated translation (Fig. 4). Hsp27 is a member of the large heat shock protein (HSP) families that are ubiquitously expressed in many organisms in both prokaryotes and eukaryotes. The function of Hsp27 is to prevent the protein aggregation during the heat shock and protect cells from cellular stress such as pathogen invasion. EV-A71 infection up-regulates the protein expression of Hsp27, which can activate viral 2A^pro^ to promote viral IRES-mediated translation [53] (Fig. 4). Hsc70 is a widely expressed cellular protein located in both the nucleus and cytoplasm [62]. The important role of Hsc70 is to regulate clathrin-mediated endocytosis. Hsc70 regulates the entry of EV-A71 and Japanese encephalitis virus (JEV) into host cells by endocytosis [63, 64] (Fig. 4). After EV-A71 infection, Hsc70 also enhances viral 2A^pro^ activity to promote viral IRES activity [54]. DDX3 is a member of the DEAD-box RNA helicase family. DDX3 is known to be involved in the regulation of mRNA translation and cell cycle [65, 66]. In addition, DDX3 is also implicated in controlling viral infections, such as JEV, HBV, HCV, and human immunodeficiency virus type 1 (HIV-1). It was recently reported that DDX3 is required for stimulation of EV-A71 IRES activity [42]. Through interaction with the C-terminal cleavage fragment of eIF4G, DDX3 may be recruited to a region near domain VI of EV-A71 IRES and then unwind the secondary structure to facilitate ribosome entry [42] (Fig. 4).

**Fig. 5:**
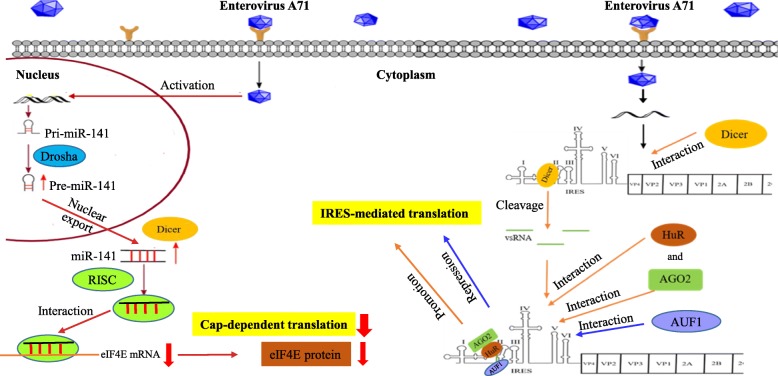
The regulatory roles of miR-141 and vsRNA in EV-A71 translation. EV-A71 infection upregulates miR-141 expression through activation of EGR1 transcription factor. miR-141 targets the 3′ UTR of eIF4E mRNA to inhibit eIF4E protein expression, and thus inhibits cap-dependent translation of host mRNAs. After EV-A71 infection, Dicer cleaves EV-A71 IRES to produce vsRNAs, which repress IRES-mediated translation and virus replication. vsRNAs may alter the binding of AUF1, Ago2, and/or HuR to IRES, and thus downregulate or upregulate viral IRES-mediated translation

MicroRNAs are small, non-protein-encoded RNAs that interfere with the normal function of endogenous mRNA. By post-transcriptional regulation of gene expression, miRNAs are also affected by viruses to promote viral infections; such as certain cellular miRNAs that regulate HCV and HIV-1 replication. EV-A71 infection also activates the transcription factor EGR1 to induce the expression of miR-141, which targets the cap-binding protein eIF4E to shut off host protein synthesis [67] (Fig. 5). Up-regulation of miR-141 may facilitate the conversion from cap-dependent to cap-independent translation, thereby promoting viral propagation. Viral infection can induce the production of virus-derived small RNAs (vsRNAs). After EV-A71 infection, Dicer cleaves viral RNA to produce at least four vsRNAs [68] (Fig. 5). One of these vsRNAs, vsRNA1, derived from the domain II of EV-A71 IRES, reduces IRES activity and virus replication [43]. The mechanism of vsRNA1 action remains unclear.AU-rich element binding factor 1 (AUF1), an mRNA decay factor, interacts with the EV71 IRES to negatively regulate IRES-mediated translation [69]. HuR is a member of the ELAVL protein family and its well-known function is to stabilize mRNA in order to regulate gene expression. HuR and the RISC subunit Argonaute 2 (Ago2) were identified as two ITAFs that bind to the domain II of EV-A71 IRES to promote IRES activity and virus replication [43]. AUF1, HuR and Ago2 associate with the same IRES domain (domain II) in EV-A71 [43]. It is speculated that vsRNA1 might alter the binding of AUF1, Ago2, and/or HuR to regulate viral IRES-mediated translation (Fig. 5).

## Conclusions

Despite the progress in our understanding of the EV-A71 translation in the last 20 years, many questions remain on such basic aspects as how the viral genome is translated efficiently. Also, the interactions between EV-A71 and host cellular factors on the translational machinery remain either controversial or poorly known. As we have known that EV-A71 manipulates the host cell translation devices, using an elegant RNA-centric strategy in infected cells, therefore, viral RNA plays an important role in controlling the stage of protein synthesis. In this review, we also describe how EV-A71 utilizes protein factors and small RNAs in the host to promote viral IRES-mediated translation rather than host cell cap-dependent translation. Such aspects could become critical in our understanding of EV-A71 viral pathogenesis. Hopefully, more basic research in the future will give us a clearer understanding of the translation of EV-A71 and take the opportunity to find more ways to fight against the virus.

## Data Availability

Not applicable.
